# MicroRNA-721 regulates gluconeogenesis via KDM2A-mediated epigenetic modulation in diet-induced insulin resistance in C57BL/6J mice

**DOI:** 10.1186/s40659-024-00495-0

**Published:** 2024-05-14

**Authors:** Shaheen Wasil Kabeer, Shivam Sharma, Shalemraju Sriramdasu, Kulbhushan Tikoo

**Affiliations:** Laboratory of Epigenetics and Diseases, Department of Pharmacology and Toxicology, National Institute of Pharmaceutical Education and Research, Sector-67, S.A.S. Nagar, Punjab, 160062 India

**Keywords:** Insulin resistance, Gluconeogenesis, Epigenetic regulation, miR-721, FOXO1

## Abstract

**Background:**

Aberrant gluconeogenesis is considered among primary drivers of hyperglycemia under insulin resistant conditions, with multiple studies pointing towards epigenetic dysregulation. Here we examine the role of miR-721 and effect of epigenetic modulator laccaic acid on the regulation of gluconeogenesis under high fat diet induced insulin resistance.

**Results:**

Reanalysis of miRNA profiling data of high-fat diet-induced insulin-resistant mice model, GEO dataset (GSE94799) revealed a significant upregulation of miR-721, which was further validated in invivo insulin resistance in mice and invitro insulin resistance in Hepa 1–6 cells. Interestingly, miR-721 mimic increased glucose production in Hepa 1–6 cells via activation of FOXO1 regulated gluconeogenic program. Concomitantly, inhibition of miR-721 reduced glucose production in palmitate induced insulin resistant Hepa 1–6 cells by blunting the FOXO1 induced gluconeogenesis. Intriguingly, at epigenetic level, enrichment of the transcriptional activation mark H3K36me2 got decreased around the FOXO1 promoter. Additionally, identifying targets of miR-721 using miRDB.org showed H3K36me2 demethylase KDM2A as a potential target. Notably, miR-721 inhibitor enhanced KDM2A expression which correlated with H3K36me2 enrichment around FOXO1 promoter and the downstream activation of the gluconeogenic pathway. Furthermore, inhibition of miR-721 in high-fat diet-induced insulin-resistant mice resulted in restoration of KDM2A levels, concomitantly reducing FOXO1, PCK1, and G6PC expression, attenuating gluconeogenesis, hyperglycemia, and improving glucose tolerance. Interestingly, the epigenetic modulator laccaic acid also reduced the hepatic miR-721 expression and improved KDM2A expression, supporting our earlier report that laccaic acid attenuates insulin resistance by reducing gluconeogenesis.

**Conclusion:**

Our study unveils the role of miR-721 in regulating gluconeogenesis through KDM2A and FOXO1 under insulin resistance, pointing towards significant clinical and therapeutic implications for metabolic disorders. Moreover, the promising impact of laccaic acid highlights its potential as a valuable intervention in managing insulin resistance-associated metabolic diseases.

**Graphical Abstract:**

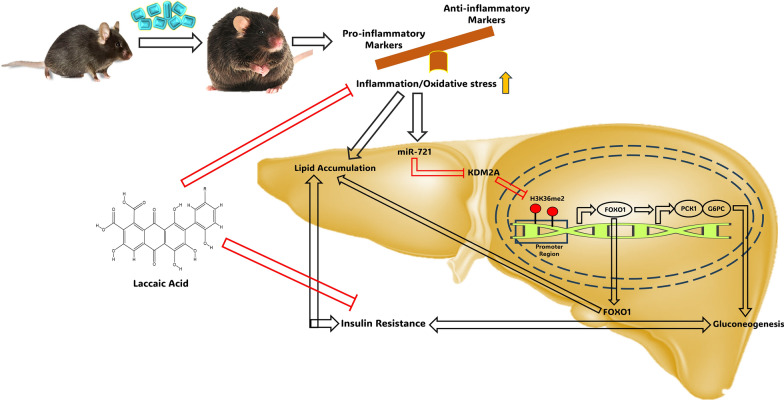

**Supplementary Information:**

The online version contains supplementary material available at 10.1186/s40659-024-00495-0.

## Background

Abnormally increased gluconeogenesis plays a central role in the development and progression of insulin resistance and subsequent metabolic disorders [1]. Unravelling the intricate molecular mechanisms that underlie these phenomena is essential for the development of effective therapeutic approaches. Hepatic glucose metabolism is strictly regulated by hormones, particularly glucagon and insulin [2]. Insulin tightly regulates hepatic gluconeogenesis via direct suppressive effects on the liver and indirect suppressive actions through its effects on other tissues like pancreatic β-cells, adipose tissue, skeletal muscles and brain [3, 4]. However, under insulin-resistant conditions, there is an overproduction of glucose via gluconeogenesis which is known to significantly contribute to hyperglycemia, a characteristic feature of disorders associated with insulin resistance [5, 6]. Chronic inflammation resulting from insulin resistance and increased circulating free fatty acids are considered among the primary drivers of increased gluconeogenesis under insulin-resistant conditions [7]. This involves dysfunction at the level of transcription factors like Forkhead box protein O1 (FOXO1), CCAAT enhancer binding protein alpha (CEBPα), Peroxisome proliferator-activated receptor-gamma coactivator (PGC)-1alpha (PGC1α), etc. which regulate the expression of various gluconeogenic genes like Phosphoenolpyruvate carboxykinase 1 (PCK1) and Glucose 6 phosphatase catalytic subunit (G6PC) [8, 9]. Previous studies by us and others have demonstrated a marked increase in FOXO1 expression in the context of metabolic disorders [8, 10, 11]. Notably, in the context of increased global levels of H3K36me2 under diet-induced insulin resistance, these studies have underscored the crucial involvement of H3K36me2 in enhancing the FOXO1 expression [10–12].

MicroRNAs (miRNAs) are small RNA molecules that do not code for proteins, but are known to act as key regulators of gene activity and cellular function. Many miRNAs have been linked to the regulation of hepatic gluconeogenesis under both physiological and pathological conditions [13–16]. High-fat diets (HFD) are known to induce insulin resistance and trigger complex changes in cellular pathways, including alterations in miRNA expression [17]. The establishment of a chronic inflammatory state is considered among the primary mechanisms driving diet-induced insulin resistance and the associated alterations in cellular functioning [18]. A multitude of studies have demonstrated that miRNAs linked to inflammation hold promise in predicting insulin resistance associated with obesity [19, 20]. Upregulation of miR-721 has been reported in many inflammation associated conditions like acute myocarditis and insulin resistance [21, 22]. It has been reported to aggravate insulin resistance in adipocytes by targeting peroxisome proliferator-activated receptor gamma (PPARγ) [21].

Laccaic acid, structurally a polyhydroxy anthraquinone is derived from the secretions of lac insect *Kerria lacca*. It has been reported to exhibit potent DNA methyltransferase 1 (DNMT1) inhibitory, antioxidant, anti-inflammatory and anticancer properties [23–25]. We have also previously reported its potential to alleviate diet induced insulin resistance in mice via anti-inflammatory and epigenetic modulatory actions [11]. It favourably altered epigenetic landscape around FOXO1 promoter, restoring the changes in H3K27me3 and H3K36me2 levels induced by the high fat diet regimen.

In this study, we show that miR-721 is upregulated under diet-induced insulin-resistant conditions and identified Lysine-specific demethylase 2A (KDM2A) as a target of miR-721. We demonstrate that miR-721 positively regulates hepatic gluconeogenesis and we explore the signaling pathway involved. Additionally, we report that laccaic acid could attenuate insulin resistance and reduce gluconeogenesis via altering H3K36me2 methylation around the FOXO1 promoter by reducing the elevated miR721 and restoring KDM2A/FOXO1 axis.

## Materials and methods

### Cell culture

Hepa 1–6 (Murine hepatoma cell line) was acquired from National Centre for Cell Sciences (NCCS) Pune, India. DMEM high glucose media containing 10% v/v FBS, 100 U/mL penicillin, and 100 mg/mL streptomycin while maintaining a humidified environment with 5% CO2 at 37 °C was utilized for growing these cells. Insulin resistance was induced as described previously [26]. Palmitate dissolved in ethanol was added to a 20 percent FFA-free BSA (sigma) solution warmed to 37 °C. This mixture was further subjected to dilution with plain medium to a final concentration of 5 percent BSA, 1 percent ethanol and 250uM palmitate. Before adding onto the cells, the palmitate solution was cleared by heating it at 37 °C and then filter sterilised. As a control for palmitate, 5 percent BSA and 1 percent ethanol were used. Palmitate was prepared fresh for each experiment.

### Mimic/inhibitor transfection

For transfection of miR-721 mimic (Qiagen, Cat. No. 219600), inhibitor (Qiagen, Cat. No. 339121) and their respective controls (Qiagen negative control mimic Cat. No. 339173, inhibitor control Cat. No. 339121), Lipofectamine 3000 (Invitrogen, CA, USA) was utilized following the instructions provided in the kit. In brief, For 24 h, Hepa 1–6 cells were cultured in 12-well or 6-well plates in antibiotic free medium. In a serum-free OptiMEM medium, 50 nM of miR-721 mimic, scramble mimic, or miR-721 inhibitor, scramble inhibitor, and Lipofectamine 3000 reagent was added independently for each well. These above mentioned components were subjected to room temperature for 5 min followed by mixing and incubation for 20 min. The resulting transfection mix was subsequently added to cells in complete growth medium and left to incubate for 24 h. On completion of incubation, the media was removed, followed by subsequent washing with PBS. Fresh media was added and the cells were used for further experiments.

### Insilico prediction of miR-721 targets

Multiple target prediction software tools were utilized in this study to identify the potential targets of miR-721. These tools included miRDB (http://mirdb.org), TargetScan (http://www.targetscan.org), miRWalk (http://mirwalk.umm.uni-heidelberg.de), miRmap (http://mirmap.ezlab.org), and StarMir (http://www.starmirdb.org). Each software employs distinct algorithms and databases to predict miRNA-target interactions based on sequence complementarity, evolutionary conservation, and other relevant features. Consensus predictions from these multiple tools were further analyzed and KDM2A was selected as a potential target gene for experimental validation.

### Invitro glucose production

Invitro glucose production was estimated as per the previously reported protocol [27]. The cells subcultured into 12-well plates were subjected to different treatments. Before the termination of incubation, the media was changed to glucose-free DMEM supplemented with 20 mM lactate (Sigma) and 2 mM pyruvate (SRL chemicals, India), and the cells were incubated in this medium for 4 h. After 4 h, the glucose concentration in the medium was assessed using a glucose assay kit (Sigma). The glucose levels were adjusted relative to total protein content.

### Animal study

Four to six weeks old male C57BL/6 J mice, procured from the Small Animal Facility for Experimentation (SAFE), IISER, India, were housed in Central Animal Facility (CAF), NIPER, India. The mice were accommodated in a controlled environment with a temperature of 22 ± 2℃, humidity at 50 ± 10%, and a 12-h light/dark cycle. They were provided unrestricted access to food and water. Each cage housed 4–6 animals, and acclimatization was conducted a week prior to the commencement of the experiment. The Institutional Animal Ethics Committee of NIPER provided approval for the study protocol (IAEC/21/09-ext1).

In total, 24 animals were divide into 2 groups, the first group consisted of six animals and the remaining 18 animals constituted the second group. The first group was fed a standard pellet diet (C 0400, Altromin, Germany) whereas the second group was fed with high fat diet (D14292, Research Diets, USA) for 12 weeks. On completion of 12 weeks, the development of insulin resistance was confirmed by determining fasting plasma glucose, cholesterol triglycerides, and performing IPGTT (intraperitoneal glucose tolerance test), ITT (insulin tolerance test) and PTT (pyruvate tolerance test). Afterwards, the HFD fed group was further divide into 3 groups of 6 animals each- HFD + SI (Scramble inhibitor), HFD + MI (miR-721 inhibitor) and HFD + LA200 (Laccaic acid) groups. The scramble and miR-721 inhibitors were administered intravenously via tail vein, twice weekly for 2 weeks at 5 nmol/dose/animal and laccaic acid (Catalog #L0095, TCI chemicals, India) was administered orally at a dose of 200 mg/kg/day for 4 weeks, suspended in 0.1% CMC.

### Biochemical parameters

Blood was collected from the retro-orbital plexus of the mice and transferred to EDTA-containing microcentrifuge tubes. The biochemical parameters were then evaluated using the plasma separated by centrifugation of the collected blood at 7000 rpm for 10 min at 4 °C. Plasma glucose, triglycerides, and total cholesterol were measured using commercially available kits from Accurex Biomedical Pvt. Ltd in Mumbai, India, in accordance with the manufacturer's instructions.

### Intraperitoneal glucose tolerance test (IPGTT), Insulin tolerance test (ITT) and Pyruvate tolerance test (PTT)

To evaluate glucose uptake capability, insulin sensitivity and glucose production, Intraperitoneal Glucose Tolerance Tests (IPGTT), Insulin Tolerance Tests (ITT), and Pyruvate Tolerance Tests (PTT) were performed following previously described protocols [28]. The animals were fasted for 12 h before IPGTT and PTT and 4 h before ITT. Subsequent to fasting, a glucose load of 2 g/kg; i.p, sodium pyruvate 2 g/kg; i.p or insulin (Humulin-R, Eli Lilly) 0.75 IU/kg; i.p, in IPGTT, PTT, and ITT, respectively. An Accu-Chek Active glucometer measured blood glucose levels at 0, 15, 30, 60, and 120 min after administering glucose, pyruvate, or insulin. The tail snip method was utilized for blood sampling. Area under the curve was determined using the changes in plasma glucose concentrations over time plot via GraphPad Prism 8 software.

### Histopathology

Histopathological assessment was conducted following earlier reported procedures [11]. Briefly, the animals were euthanized and liver was dissected and fixed using 10% neutral buffered formalin, gradually dehydrated using ethanol, cleared using xylene, and finally embedded in paraffin. Afterwards, 5 μm sections were taken and air dried overnight. Deparaffinization of sections with xylene followed by rehydration with a combination of alcohol and water were stained using Haematoxylin and Eosin (H&E) and Periodic-acid Schiff (PAS). Finally, the sections were mounted using DPX mounting media and microscopic examination was carried out using an Olympus BX51 microscope (Tokyo, Japan).

For oil red O staining, frozen tissues were embedded in the OCT (optimal cutting temperature compound, Sigma) embedding medium and 5um sections were cut using cryotome (Leica CM 1860, Leica Biosystem). The sections were air dried for 1 h, fixed with neutral buffered formalin. After staining with oil red O for 20 min, the sections were counterstained with hematoxylin and mounted using glycerol gelatin mountant and examined under microscope (OLYMPUS BX51, Tokyo, Japan).

### Immunohistochemical evaluation

Immunohistochemical studies were performed in accordance with the instructions provided with the kit (ImmPRESS® Excel Amplified polymer staining kit, Antirabbit IgG, Cat# MP-7601–15, Vector Laboratories, CA, USA). Primary antibodies against KDM2A (A18636, ABclonal Technology, MA USA) and FOXO1 (A2934, ABclonal Technology, MA USA) were used. Following counterstaining with hematoxylin, the sections were mounted with DPX and observed using a microscope (OLYMPUS BX51, Tokyo, Japan). Subsequently, the images were quantified utilizing ImageJ software (NIH, MD, USA).

### RNA isolation and qRT-PCR

Liver tissue was processed to extract total RNA employing the Trizol method. Subsequently, cDNA was synthesised utilizing the Verso cDNA synthesis kit (AB1453A-Thermo Scientific) in accordance with the kit instruction manual. NanoDrop 1000 (ThermoFisher Scientific). was used to analyze the quality of the extracted RNA. Kdm2a, Foxo1, G6pc, and Pck1 expression levels were analyzed employing qRT-PCR using Brilliant III SYBR Master Mix (Agilent, Santa Clara, USA) along with gene-specific primer sequences (Eurofins India Pvt Ltd). Each gene's primer sequences are provided in Table 1. Using specific primers, actin levels were also amplified and used for normalization. 2-ΔΔCt method was used to analyze the amplification curves.

**Table 1:** List of primers used in qRT-PCR

Gene	Forward primer (5′……..3′)	Reverse primer (5′……..3′)
Foxo1	TTTTCAGCCTTGAGCAGCCT	ACTGGGAAACACCGATGGAC
Pck1	TTGAACTGACAGACTCGCCC	GGCACTTGATGAACTCCCCA
G6pc	CCTTGGTGACTGTCCTCCTG	AAGTGCTTGGTGTGGGTGAA
Kdm2a	GCCAAGGCACTTGAAAGAAA	AGCAGCCTCGAACACTCATT
Actin	CATCACCAACTGGGACGAC	ATACATGGCAGGCACGTTGA

MiRNA was extracted from Hepa 1–6 and liver tissues employing the HiPure miRNA isolation kit (Catalog #05080576001, Roche, Basel, Switzerland), and its purity was assessed using the NanoDrop 1000 spectrophotometer. The miRCURY LNA RT Kit (Catalog #339340, Qiagen, USA) and Thermal Cycler 2720 (Applied Biosystems, CA, USA) were used for cDNA synthesis. Real-time PCR analysis was conducted on an AriaMx instrument (Agilent Technologies, Inc, CA, USA) employing pre-made primer mix specific for mmu-miR-721 (Catalog #339306, Qiagen). The levels of miRNA expression were normalized against U6 small nuclear RNA (Catalog #203907). Amplification curves were analyzed using the 2-ΔΔCt method, and the resulting relative values were plotted.

### Immunoblotting

Immunoblot analyses were conducted following established procedures [10]. In brief, Hepa 1–6 cells or liver tissues underwent lysis using NP-40 lysis buffer, and the resulting protein samples were separated on 8–14% SDS-PAGE gel electrophoresis. Transblot SD Semi-Dry Transfer Cell (Bio-Rad Laboratories) was used to transfer proteins onto Polyvinylidene difluoride (PVDF) membranes which were then subjected to incubation with primary antibodies (diluted at 1:1000): KDM2A (A18636, ABclonal Technology, MA USA), FOXO1 (A2934, ABclonal Technology, MA USA), PCK1 (A22172, ABclonal Technology, MA USA), G6PC (A20193, ABclonal Technology, MA USA), and Actin (sc-47778, Santa Cruz Biotechnology, USA). Following an overnight incubation at 4℃, subsequent incubation of membranes was done with secondary antibodies conjugated with horseradish peroxidase (HRP) (Jackson Immunoresearch, USA) for 1 h at room temperature. The resulting immune complexes were detected and visualized using an enhanced chemiluminescence substrate (Invitrogen, CA, USA) and images were captured using Image Quant 500 (Amersham). Densitometric analysis of the immunoblot images was performed using ImageJ software, NIH, USA.

### Chromatin immunoprecipitation

The Chromatin Extraction kit’s (Abcam, USA, Cat. # ab117152) was used to extract chromatin from Hepa 1–6 cells and liver tissues, and ChIP-qPCR was conducted using a one-step ChIP kit (Abcam, USA, Cat.# ab117138) following the manufacturer's instructions. In summary, Hepa 1–6 cells were treated with neutral buffered formalin for cross-linking. Following cross-linking, the cells were homogenised using the lysis buffer supplied with the kit. The liver samples were minced on ice before crosslinking and proceeding further. The resulting homogenate was centrifuged, yielding a nuclear pellet, which was resuspended in chromatin extraction buffer for isolating chromatin. The fragmentation of isolated chromatin was done using BioRuptor (Diagenode) set at sonication for 10 cycles, with each cycle comprising 10 s followed by 15-s intervals. A portion (10%) of the fragmented chromatin was retained as an input control, while the rest was incubated overnight at 4℃ with the anti-H3K36me2 antibody for immunoprecipitation. Afterwards the antibody-protein-DNA complexes were washed to remove any unbound antibodies. Subsequently, the cross-linking between the proteins and DNA was reversed by treating the complexes with the DNA release buffer. The DNA thus obtained was subjected to real-time PCR analysis. Additionally, DNA from the input samples was extracted and used for normalisation. Specifically designed primers for the FOXO1 promoter region (forward: 5′—AAGTGAGATTCCCGTGGCAG -3′, reverse: 5′—GTAACCGCTTCCCACCCTAC -3') were employed to quantify the abundance of H3K36me2.

### Statistical analysis

The experimental results are presented as the mean values with standard deviations (mean ± SD). Two-tailed Student's t-test was applied to compare means of two groups. In cases where multiple groups were compared, one-way analysis of variance (ANOVA) was used and Tukey's test was employed for post-hoc analysis. A significance threshold of p < 0.05 was considered as statistically significant.

## Results

### Hepatic miR-721 is upregulated in high-fat diet-induced insulin resistance

**Fig. 1:**
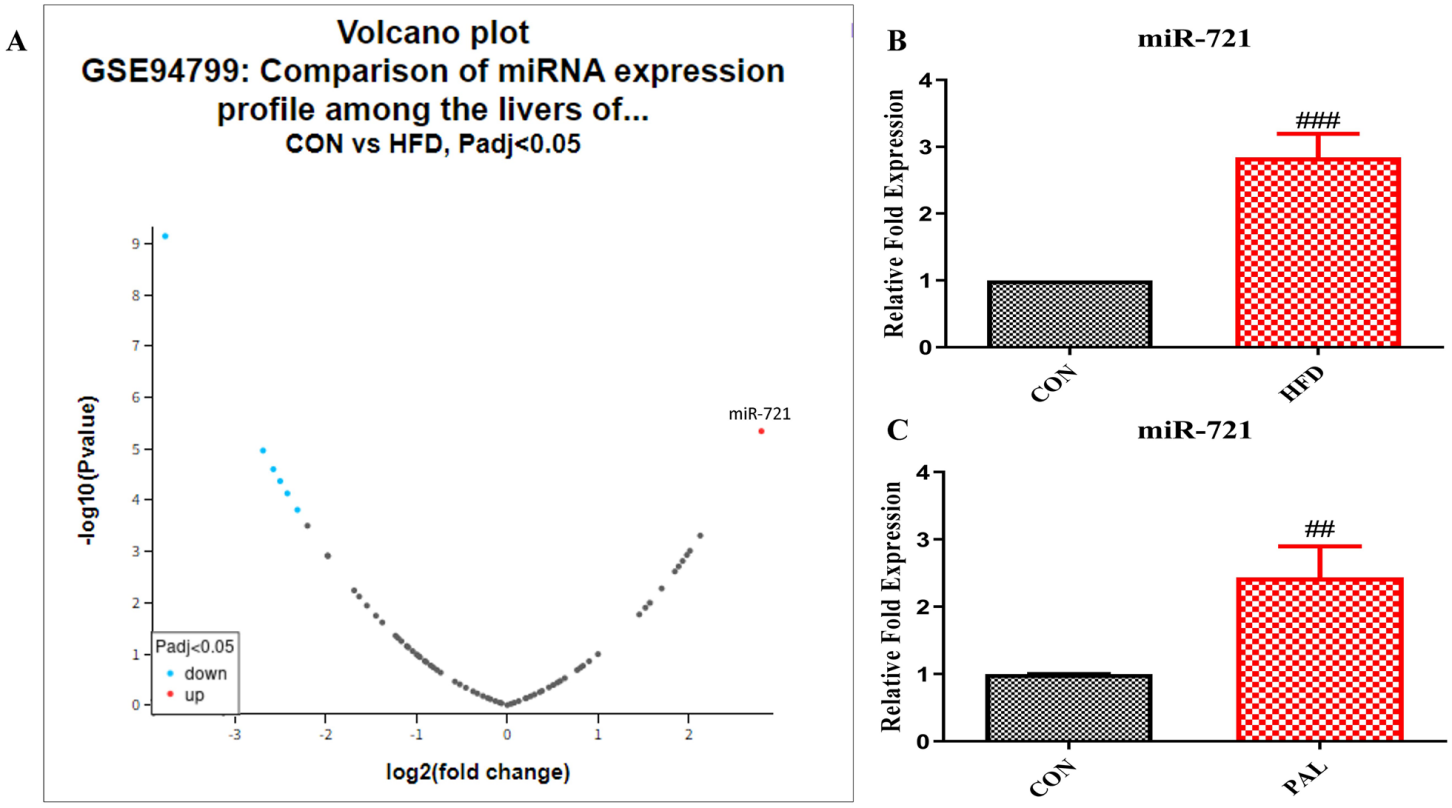
MiR-721 is upregulated under insulin resistant conditions. The figure shows volcano plot derived from GEO2R analysis of GEO dataset GSE94799 (**A**), miR-721 expression in diet induced insulin resistant mice (**B**) and miR-721 expression in palmitate induced insulin resistant Hepa 1–6 cells (**C**). Data are expressed as mean ± SD, (n = 3) ##p < 0.01, ###p < 0.001, (#) vs CON

To investigate specific hepatic miRNAs associated with high-fat diet-induced insulin resistance, we searched the literature and considered a GEO dataset (GSE94799) comprising of miRNA expression profiles of liver from control chow and high-fat diet-fed C57BL/6J mice for reanalysis of differentially expressed miRNAs. Interestingly, the volcano plot derived from the GEO2R analysis (at default parameter settings) of the GSE94799 dataset (Fig. 1A) indicated that seven miRNAs were differentially expressed in the livers of high-fat diet-fed animals. Intriguingly, miR-721 was significantly upregulated (log2 fold change-2.8) in high-fat diet-fed animals. In support of the GEO2R analysis, levels of miR-721 analyzed by rt-PCR also revealed a profound increase (≈threefold) in the expression of miR-721 in livers of high-fat diet-induced insulin-resistant mice as well as palmitate-induced insulin-resistant Hepa 1–6 cells (Fig. 1B, C). Taken together, these results further confirm that increased level of miR-721 expression is a potential marker of hepatic insulin resistance.

### miR-721 increases glucose production in Hepa 1–6 cells by regulating the gluconeogenesis pathway

**Fig. 2:**
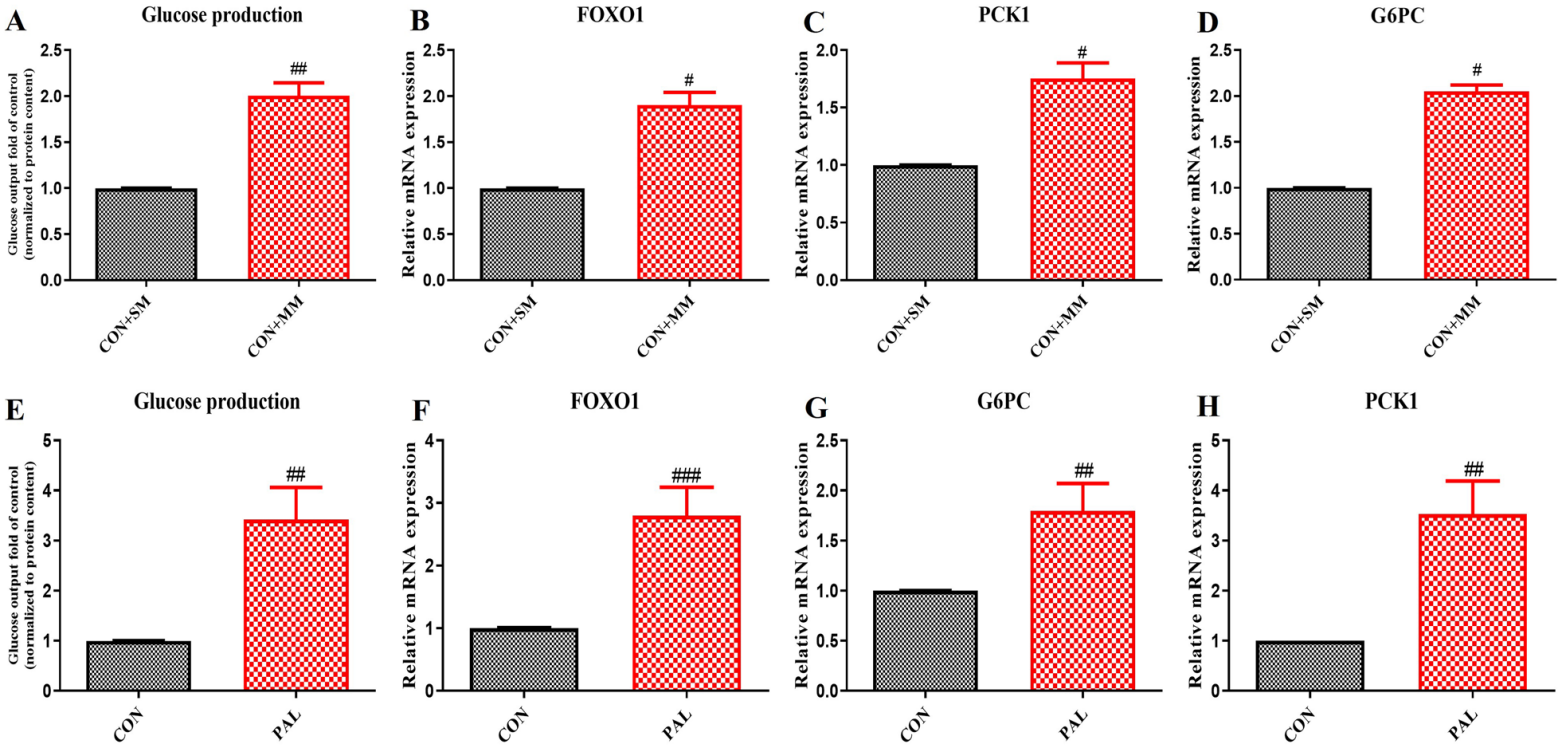
miR-721 enhances glucose production in Hepa 1–6 cells. Cells were transfected with miR-721 mimic or treated with palmitate for 24 h and glucose production was evaluated. The figure shows glucose production (**A**, **E**), expression of FOXO1 (**B**, **F**), PCK1 (**C**, **G**) and G6PC (**D**, **H**) in response to miR-721 mimic and palmitate. Data are expressed as mean ± SD, (n = 3) #p < 0.05, ##p < 0.01, ###p < 0.001, (#) vs CON. (SM-Scramble mimic, MM-miR-721 mimic)

To investigate the effect of miR-721 on glucose metabolism, we transfected murine Hepa 1–6 cells with miR-721 mimic. 24 h post-transfection, there was significant increase in glucose production as indicated by the glucose production assay (Fig. 2A). Qrt-PCR analysis of the expression of gluconeogenic genes Foxo1, Pck1 and G6pc in response to the miR-721 mimic also showed significant increase (Fig. 2B–D). Furthermore, palmitate-induced insulin resistance in Hepa 1–6 was also associated with increase in glucose production (Fig. 2E) and elevated expression of genes involved in gluconeogenesis- Foxo1, Pck1 and G6pc (Fig. 2F–H). These results suggest a detrimental role of miR-721 in gluconeogenesis.

### miR-721 regulates gluconeogenesis via targeting KDM2A

**Fig. 3:**
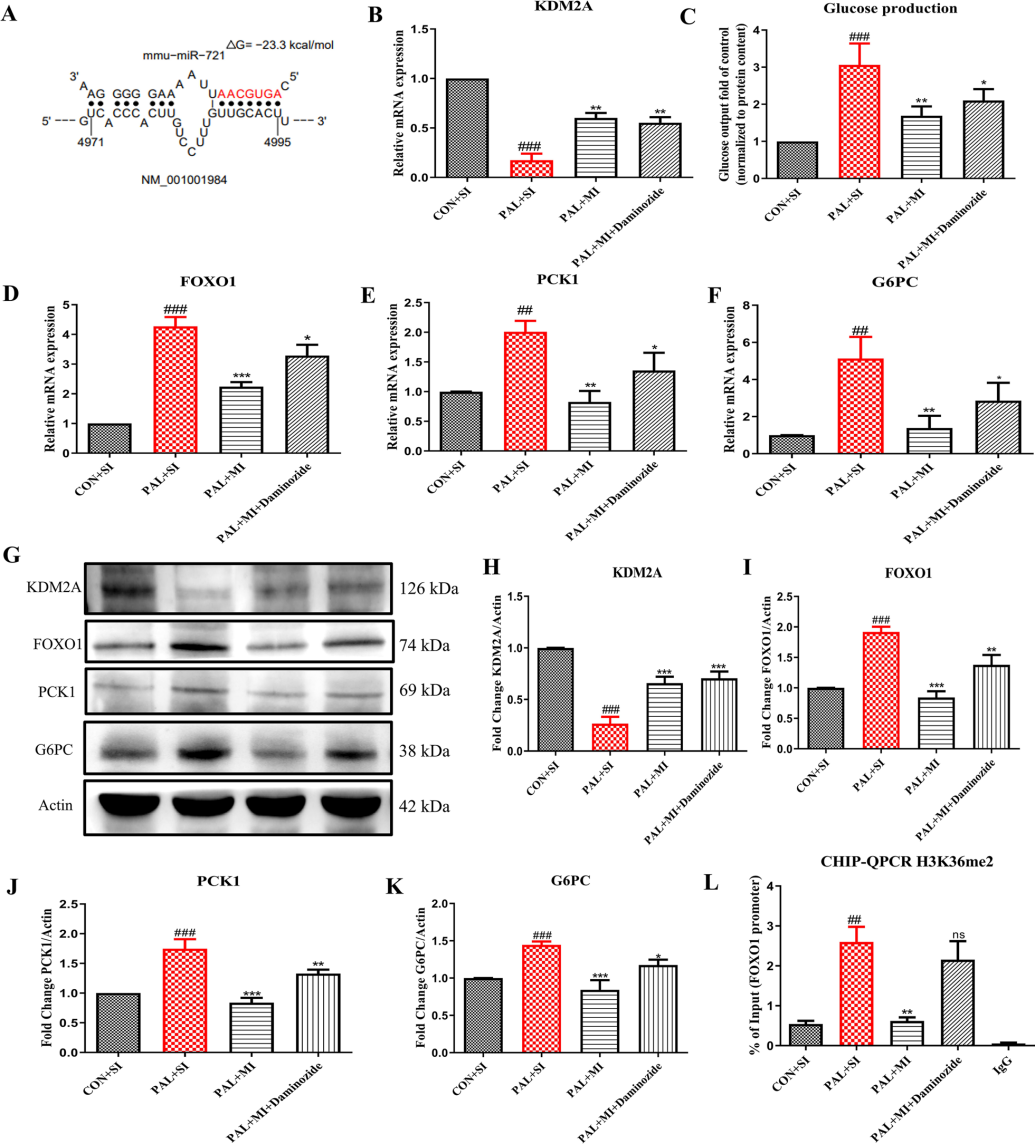
miR-721 affects gluconeogenesis via KDM2A/FOXO1 pathway. Bioinformatic analysis identified KDM2A as a potential target and to further validate the effects of miR-721 inhibition on KDM2A, Hepa 1–6 cells were transfected with miR-721 inhibitor and treated with daminozide a KDM2A inhibitor and effects on glucose production were evaluated. The figure shows the binding pose and binding free energy for miR-721 and KDM2A mRNA predicted by StarMir (**A**), mRNA expression of Kdm2a (**B**), glucose production (**C**), mRNA expression of Foxo1, Pck1, G6pc (**D**–**F**), immunoblots and quantification of KDM2A, FOXO1, PCK1, G6PC (G-K) and CHIP-qPCR of H3K36me2 for enrichment on FOXO1 promoter (L). Data are expressed as mean ± SD, (n = 3) *p < 0.05, ## or **p < 0.01, ### or ***p < 0.001, (#) vs CON and (*) vs PAL + SI group (SI: Scramble inhibitor and MI: miR-721 inhibitor)

We used various bioinformatics tools like miRDB, miRmap, miRWalk, targetscan and miRWalk to predict the targets of miR-721. Among the putative targets, we found KDM2A to be a consistently high scoring target of miR-721 across all the prediction algorithms (Additional file 1: Fig. S2). To predict the binding interaction of KDM2A mRNA transcript with miR-721 and the minimum free energy (mfe) we used the tool StarMir. The miR-721 sequence complementary to the 3′UTR region of KDM2A formed a hybrid with a minimum free energy (mfe) of − 23.3 kcal/mol (Fig. 3A). To consolidate on the role of miR-721 in regulating KDM2A, we treated Hepa 1–6 cells with miR-721 inhibitor and exposed these cells to palmitate for 24 h in the presence or absence of 10uM daminozide (a known inhibitor of KDM2A). Treatment with miR-721 inhibitor significantly reduced KDM2A expression which was not significantly affected by daminozide (Fig. 3B). miR-721 inhibition significantly reduced glucose production in palmitate-treated cells while this effect was blunted by daminozide a known KDM2A inhibitor [29] (Fig. 3C). Also, the expression of FOXO1, PCK1 and G6PC was reduced by miR-721 inhibitor while as the expression of KDM2A was restored and daminozide partially reversed these effects (Fig. 3D–F, G–K). Daminozide itself had minimal effect on the expression of KDM2A (Fig. 3G, H). Interestingly, our ChIP experiments revealed that palmitate-induced insulin resistance increased the enrichment of H3K36me2 around FOXO1 promoter along with reduced expression of Kdm2a, which was restored by miR-721 inhibition (Fig. 3L). Taken together, these observations indicate that miR-721 regulates gluconeogenesis via KDM2A/FOXO1 axis.

### mir-721 inhibition and laccaic acid improve glucose metabolism and liver histopathology in high-fat diet-induced insulin resistance

**Fig. 4:**
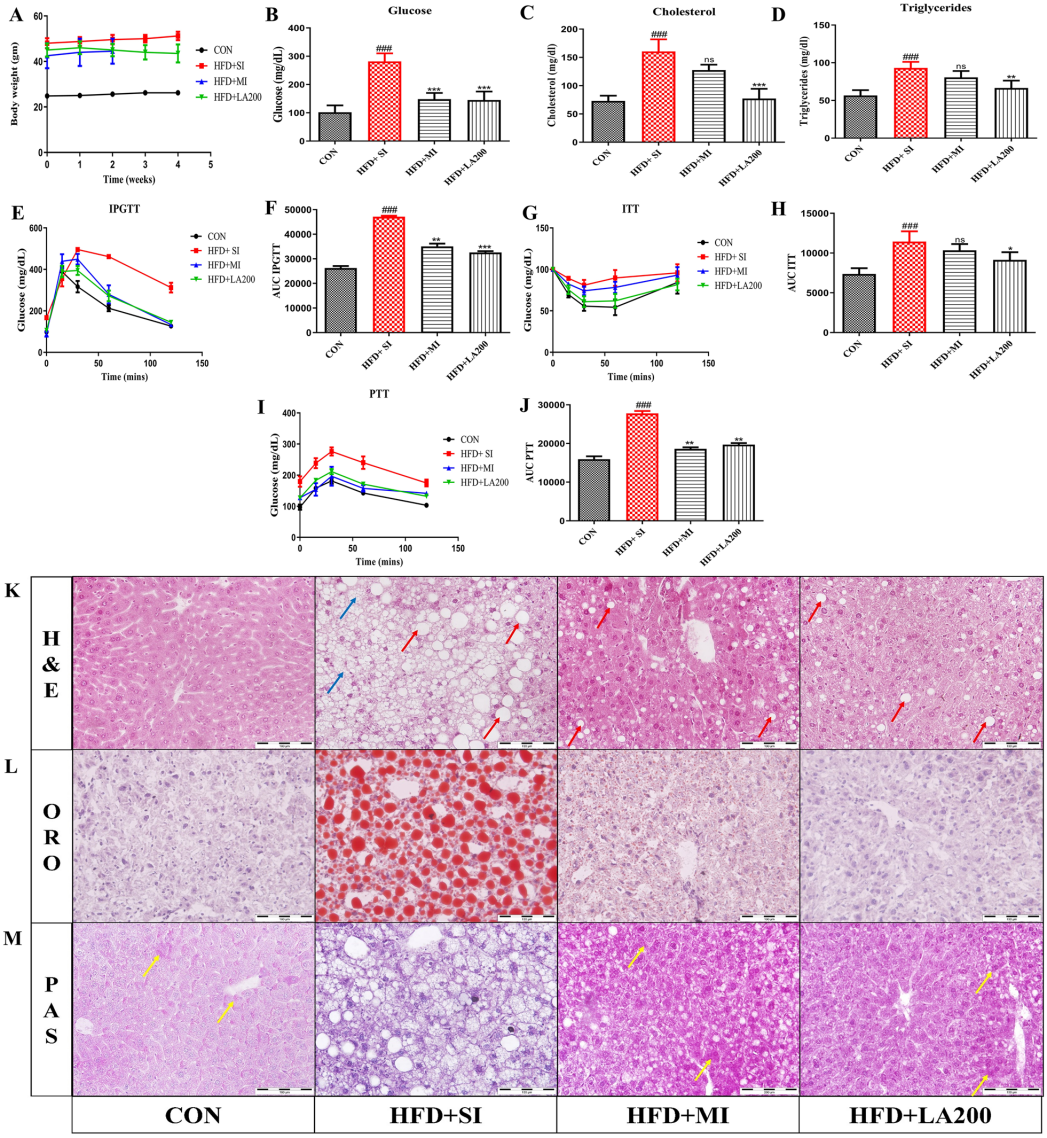
miR-721 inhibition and laccaic acid treatment improves glucose metabolism and liver histopathology under diet induced insulin resistant conditions. After administering miR-721 inhibitor and laccaic acid, blood samples were taken for biochemical estimations, and tests for glucose insulin and pyruvate were performed. Afterwards, animals were sacrificed and livers were subjected to histopathological assessment. The figure shows bodyweight (**A**), plasma glucose (**B**), total cholesterol (**C**), triglycerides (**D**), IPGTT (**E**), AUC of IPGTT (**F**), ITT (**G**), AUC of ITT (**H**), PTT (**I**), AUC of PTT (**J**), representative photomicrographs of liver H&E (**K**), oil red O staining (**L**) and PAS staining (**M**). Data are expressed as mean ± SD, (n = 3–6) *p < 0.05, ## or **p < 0.01, ### or ***p < 0.001, (#) vs CON and (*) vs HFD + SI group

To extend our in-vitro findings to a physiological context and explore the impact of miR-721 inhibition on hepatic gluconeogenesis under conditions of high-fat diet-induced insulin resistance, we developed the insulin resistance model by feeding high-fat diet to C57BL/6J mice for 12 weeks. In our previous study we observed laccaic acid could reduce gluconeogenesis under high fat diet-induced insulin-resistant conditions [11]. To further ascertain this, we checked whether laccaic acid altered miR-721 expression. At the completion of 12 weeks, the high-fat diet-fed animals displayed characteristic features of insulin resistance, there was a significant increase in body weight, blood glucose, triglycerides and cholesterol (Additional file 1: Fig. S2A–D). Also, the animals had reduced glucose tolerance, insulin responsiveness and an increased hepatic glucose output in response to a bolus injection of pyruvate (Additional file 1: Fig. S2E–J). Notably, treatment with miR-721 inhibitor (MI) (5nmol/dose) [4] and laccaic acid (200mg/kg), improved plasma glucose (Fig. 4B), and glucose tolerance (Fig. 4E, F) and also reduced hepatic glucose output in response to pyruvate load (Fig. 4I, J) along with improvements in plasma cholesterol (Fig. 4C), triglycerides (Fig. 4D) and insulin responsiveness (Fig. 4G, H) which was more significant in laccaic acid treated group. Additionally, bodyweight was also reduced in laccaic acid treated group (Fig. 4A). At the histopathological level, H&E staining revealed that MI and laccaic acid improved the macrovesicular steatosis and hepatocyte vacuolization (Fig. 4K), ORO staining indicated a reduction in lipid accumulation (Fig. 4L) and PAS staining showed a significant improvement in glycogen deposition in treated animals as compared to diseased group (Fig. 4M).

### miR-721 inhibition and laccaic acid reduce hepatic gluconeogenesis via improving KDM2A/FOXO1 signaling in high-fat diet-induced insulin resistance

**Fig. 5:**
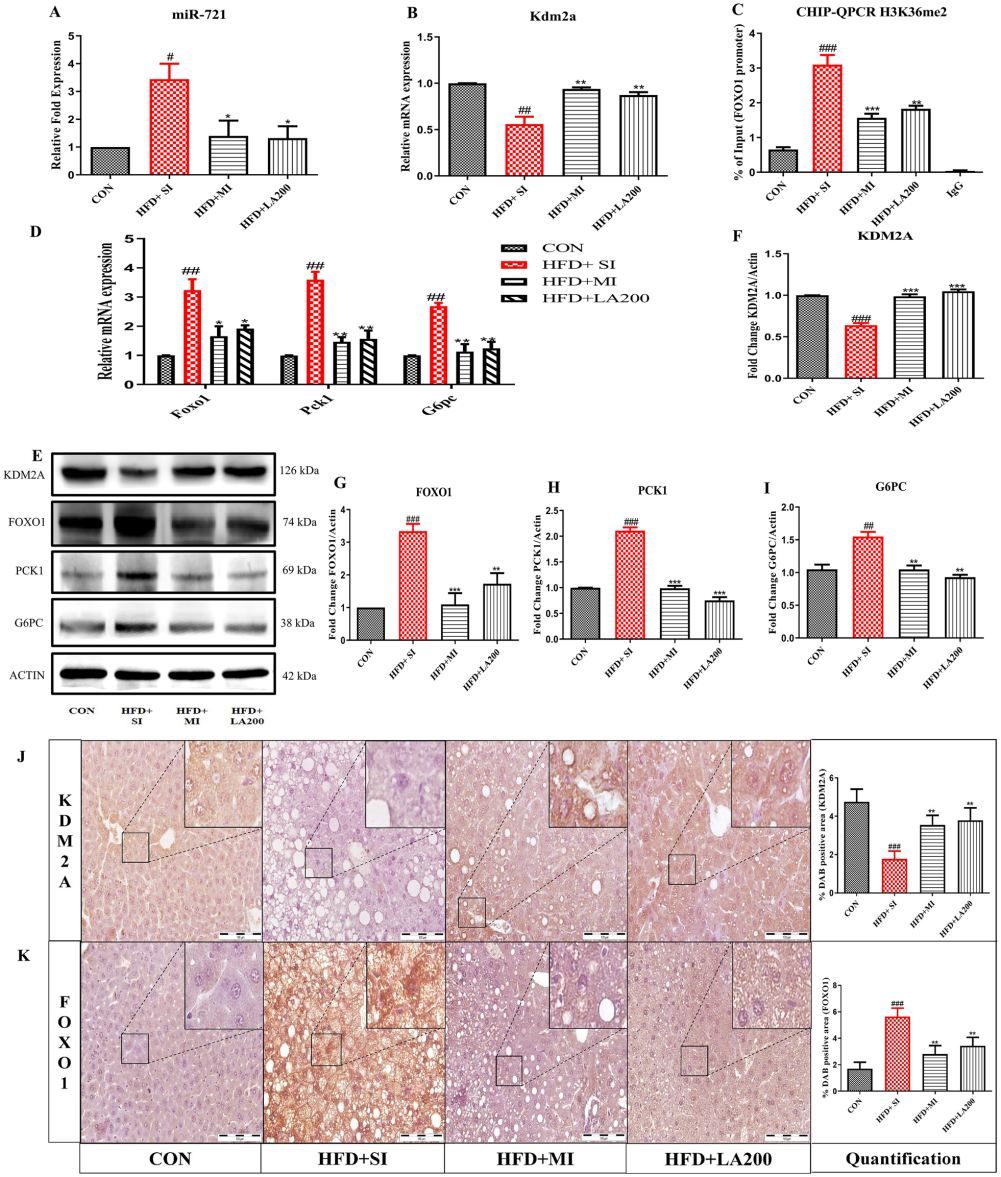
Effect of miR-721 inhibition and laccaic acid on FOXO1 regulated gluconeogenic pathway. Expression analysis was carried out in liver to investigate the effects of miR-721 inhibitor and laccaic acid on the FOXO1 and gluconeogenic pathway. The figure shows the expression of miR-721 (**A**), expression of Kdm2a (**B**), CHIP-qPCR of H3K36me2 for enrichment on Foxo1 promoter (**C**), mRNA expression of Foxo1, Pck1 and G6pc (**D**), immunoblots and densitometric analysis of KDM2A, FOXO1, PCK1 and G6PC (**E**–**I**) and representative photomicrographs of IHC of KDM2A and FOXO1 with quantification (**J**, **K**). Data are expressed as mean ± SD, (n = 3) # or*p < 0.05, ## or **p < 0.01, ### or ***p < 0.001, (#) vs CON and (*) vs HFD + SI group

Building upon our observations in the biochemical and histological features, next we explored the effects of miR-721 inhibitor and laccaic acid on the gluconeogenic pathway involving the master regulator FOXO1. There was significant upregulation of miR-721 in the high-fat diet fed group which was reduced by treatment with MI and LA (Fig. 5A). Further, we observed reduced expression of demethylating enzyme Kdm2a in the disease group in comparison to the control which was improved by LA and MI (Fig. 5B). Our ChIP experiments revealed increased enrichment of gene activation mark H3K36me2 around the Foxo1 gene promoter region in the disease group which was lost upon MI and laccaic acid treatment (Fig. 5C), which resulted in reduced expression of Foxo1 (Fig. 5D, E, G). Immunohistochemical studies also indicated improvement in the levels of KDM2A and reduction in FOXO1 levels in mice treated with MI and laccaic acid as compared to insulin resistant group (Fig. 5J, K) supporting our earlier results with laccaic acid. This all resulted in preventing the aberrant activation of the gluconeogenic pathway as indicated by decreased expression of Pck1 and G6pc (Fig. 5D, E, H, I).

## Discussion

The current study, was focused on investigating the role of differentially expressed miRNAs in high fat diet-induced insulin resistance. Performing the reanalysis of a relevant miRNA expression dataset (GSE94799) to uncover differentially expressed miRNAs revealed miR-721 as a standout candidate, showing significant upregulation in high fat diet-fed animals. This was validated in our diet-induced insulin-resistant model in mice and palmitate-induced insulin-resistant Hepa 1–6 cells. Further confirming the involvement of miR-721 in the development and progression of diet-induced insulin resistance. Multiple studies have reported that palmitate exposure in hepatocytes induces insulin resistance which is accompanied by the dysregulation of gluconeogenesis and increased glucose production [30–33]. Here, we report that miR-721 is significantly upregulated in response to palmitate treatment. Our experiments with miR-721 mimic pointed towards the role of miR-721 in regulating hepatic gluconeogenesis as indicated by increased glucose production and the activation of the FOXO1-regulated gluconeogenic pathway.

Performing bioinformatics analysis showed that one of the putative targets of miR-721 was histone demethylase enzyme-KDM2A. KDM2A is a histone demethylase that specifically demethylates H3K36me2, which is considered as a mark of active transcription sites. Multiple studies have reported that the levels of H3K36me2 are increased globally as well as around FOXO1 promoter region in response to high fat diet feeding in mice [10, 12]. In our study palmitate induced insulin resistance in hepatocytes significantly increased H3K36me2 levels on the promoter site of FOXO1. This coincided with a decrease in Kdm2a levels and increase in glucose production. Further deciphering the role of miR-721, oligonucleotide inhibitor of miR-721 restored KDM2A levels which inturn reduced H3K36me2 levels on the promoter site of FOXO1 and concomitantly reduced glucose production. Pharmacological inhibition of KDM2A by a known inhibitor, daminozide further confirmed that these effects on gluconeogenesis by miR-721 inhibition, are mediated at least partly via KDM2A [29]. In our study daminozide did not alter the expression of Kdm2a, but inhibition of KDM2A was evident from the decrease in the H3K36me2 levels around Foxo1 promoter. The mechanism of KDM2A inhibition by daminozide has been reported involve the chelation of the metal within the enzyme active site through its hydrazide carbonyl and dimethylamino groups [34]. These findings are in consonance with previous studies reporting crucial role of KDM2A in regulating hepatic gluconeogenesis by altering the levels of H3K36me2 around the promoter regions of gluconeogenic transcription factors like CEBPα and further affecting the expression of important genes involved in gluconeogenesis like Pck1 and G6pc [35].

Under invivo conditions, miR-721 inhibitor significantly improved the fasting blood glucose levels, and glucose tolerance and reduced glucose production in response to an intraperitoneal pyruvate load, indicating that miR-721 plays an important role in glucose homeostasis. Furthermore, enhanced lipid build-up, vacuolization and macrovesicular steatosis are the cardinal features displayed by the liver under high-fat diet-induced insulin-resistant conditions [36]. These histopathological alterations were attenuated by miR-721 inhibition. Various studies have reported that aberrant hepatic gluconeogenesis plays a detrimental role in increasing the accumulation of lipids in the liver under insulin-resistant conditions with FOXO1 playing a central role by enhancing lipogenesis via pathways including the activation of MTORC2/SREBP1C axis [6, 37, 38]. Therefore, the observed improvement in the hepatic lipid accumulation upon miR-721 inhibition could be attributed to its beneficial effects on glucose metabolism.

**Fig. 6:**
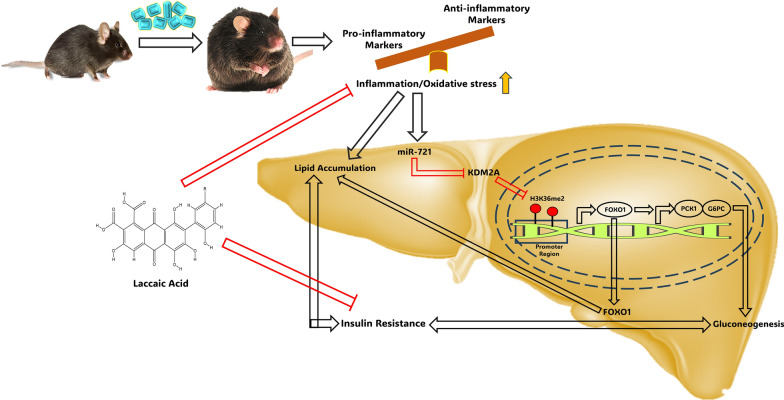
Proposed mechanism of miR-721 regulation of gluconeogenesis under high fat diet induced insulin resistance and effect of laccaic acid. miR-721 is overexpressed under diet induced insulin resistance conditions resulting in downregulation of KDM2A, which inturn increases H3K36me2 around FOXO1 promoter. Enrichment of H3K36me2 around FOXO1 increases its expression and results in the activation of the gluconeogenic program by enhancing PCK1 and G6PC expression. Laccaic acid blunts this miR-721/KDM2A/FOXO1 axis and reduces gluconeogenesis and alleviates insulin resistance

Furthermore, we have previously reported that laccaic acid, known for its anti-inflammatory properties, reduces gluconeogenesis and alleviates insulin resistance by restoring the high-fat diet induced epigenetic alterations around the FOXO1 promoter [11]. Interestingly, in the current study, laccaic acid also reduced the expression of miR-721 and decreased the expression of gluconeogenic genes via KDM2A/FOXO1 axis. Based on available literature, we hypothesize that miR-721 is involved in regulation of inflammatory response as its expression has been reported to increase under inflammatory conditions like acute myocarditis in mice models, in response to infections as well as drug induced liver injury [22, 39–41]. Ke et, al, have also reported that miR-721 expression increases upon treatment with tumor necrosis factor-alpha in adipocytes further suggesting that this miRNA is strongly associated with inflammation [21]. The association of miR-721 with inflammation, supported by a growing body of evidence could be the most plausible explanation for the observed anti-gluconeogenic activity of laccaic acid (Fig. 6). Together, these findings suggest a crucial role of miR-721 in pathogenesis of insulin resistance and project laccaic acid as a potential therapeutic avenue against insulin resistance.

## Limitations of the study

Notwithstanding the foregoing, additional experimentation such as luciferase assay are required to validate a direct interaction between miR-721 and KDM2A, to provide conclusive evidences of this relationship in addition to all bioinformatics analyses. Also, the effects of miR-721/KDM2A axis on other transcription factors involved in gluconeogenesis can be focus of further studies, which could improve our understanding of this complex physiologic process. Furthermore, the precise mechanisms of the anti-inflammatory actions displayed by laccaic acid need to be investigated.

## Conclusion

In summary, our investigation into the role of miR-721 in gluconeogenesis regulation under conditions of insulin resistance uncovers a pivotal molecular mechanism. By elucidating the intricate interplay between miR-721, KDM2A, and FOXO1, we provide valuable insights into the epigenetic landscape governing glucose homeostasis which can have potential therapeutic implications. Furthermore, our findings shed light on the potential therapeutic role of laccaic acid in preventing metabolic dysregulations associated with insulin resistance.

### Supplementary Information

**Additional file 1: Figure S1.** High fat diet feeding for 12 weeks develops insulin resistance in C57BL/6J mice. At the end of 12 weeks, animals were fasted overnight, blood was withdrawn from the retro-orbital plexus, and glucose, triglycerides, and cholesterol were estimated. For IPGTT and PTT animals were fasted overnight whereas fasting time before ITT was 4 hrs. The figure shows body weight after 12 week HFD feeding (A), plasma glucose (B), triglycerides (C), total cholesterol (D), IPGTT (E), AUC of IPGTT (F), ITT (G), AUC of ITT (H), PTT (I) and AUC of PTT (J). Data are expressed as mean ±SD. (N=6,18), *p<0.05, **p<0.01, ***p<0.001. * vs CON. **Figure S2.** Insilico analysis of mm-miR-721 targets. The figure shows (A) miR-721 target prediction analysis performed at www.informatics.jax.org/ , (B) miR-721 interaction details obtained from miRDB.org and (C) miR-721 targeting of Kdm2A as predicted by miRmap.

## Data Availability

All the data described in this study is contained within the article.

## Abbreviations

KDM2A: Lysine demethylase 2A
FOXO1: Forkhead box protein O1
PCK1: Phosphoenolpyruvate carboxykinase 1
G6PC: Glucose 6 phosphatase catalytic subunit
CEBPα: CCAAT enhancer binding protein alpha
PGC1α: Peroxisome proliferator-activated receptor-gamma coactivator 1 alpha
MTORC2: Mammalian target of rapamycin complex 2
SREBP1C: Sterol Regulatory Element Binding Protein 1c
