# Supplementary material for: MicroRNA-721 regulates gluconeogenesis via KDM2A-mediated epigenetic modulation in diet-induced insulin resistance in C57BL/6J mice
Source: Biol Res. 2024 May 14;57:27. doi: 10.1186/s40659-024-00495-0 (PMC11092102; doi:10.1186/s40659-024-00495-0)
Supplement: Supplementary file 1 — Additional file 1: Figure S1. High fat diet feeding for 12 weeks develops insulin resistance in C57BL/6J mice. At the end of 12 weeks, animals were fasted overnight, blood was withdrawn from the retro-orbital plexus, and glucose, triglycerides, and cholesterol were estimated. For IPGTT and PTT animals were fasted overnight whereas fasting time before ITT was 4 hrs. The figure shows body weight after 12 week HFD feeding (A), plasma glucose (B), triglycerides (C), total cholesterol (D), IPGTT (E), AUC of IPGTT (F), ITT (G), AUC of ITT (H), PTT (I) and AUC of PTT (J). Data are expressed as mean ±SD. (N=6,18), *p<0.05, **p<0.01, ***p<0.001. * vs CON. Figure S2. Insilico analysis of mm-miR-721 targets. The figure shows (A) miR-721 target prediction analysis performed at www.informatics.jax.org/ , (B) miR-721 interaction details obtained from miRDB.org and (C) miR-721 targeting of Kdm2A as predicted by miRmap. [file 40659_2024_495_MOESM1_ESM.docx]

**Supplementary Figure**

**
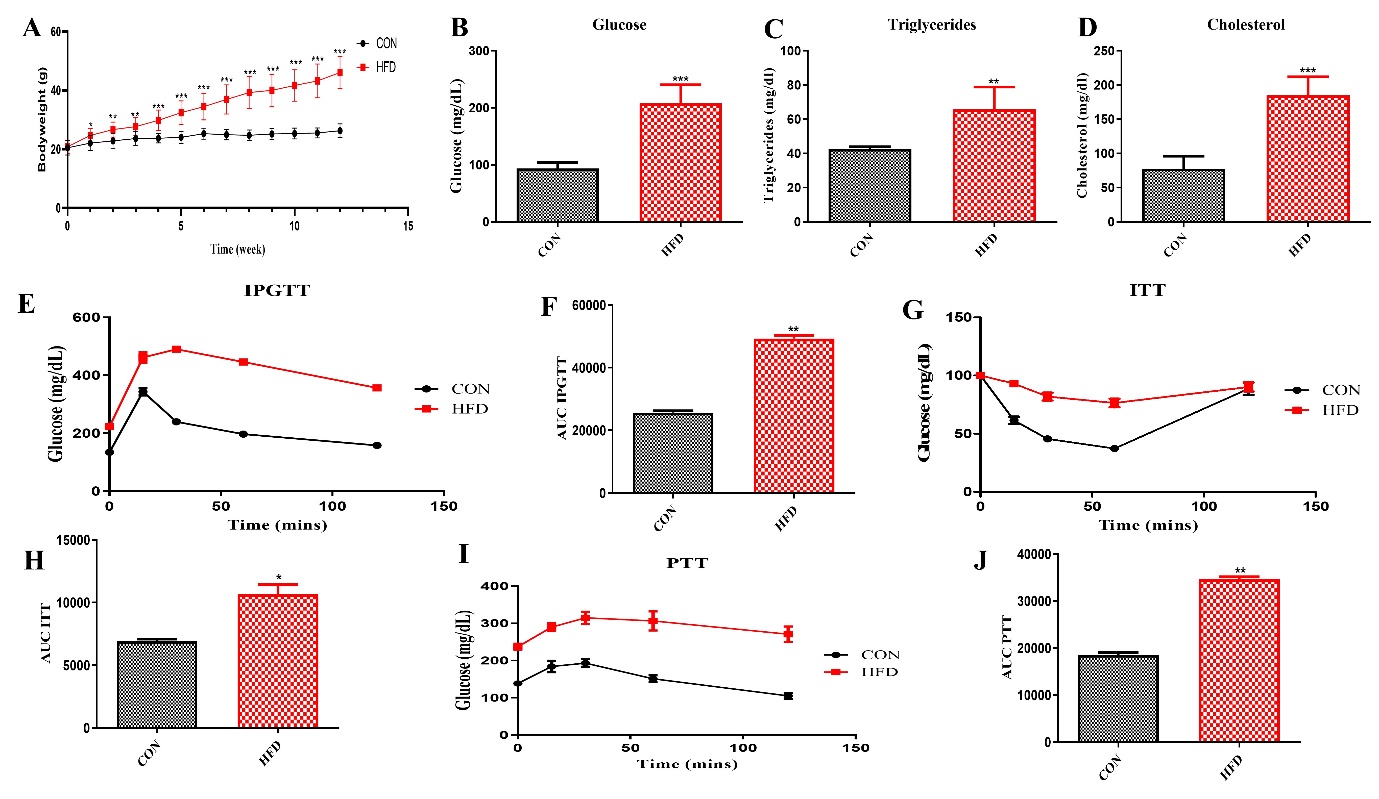
**

**Fig. S1. High fat diet feeding for 12 weeks develops insulin resistance in C57BL/6J mice.** At the end of 12 weeks, animals were fasted overnight, blood was withdrawn from the retro-orbital plexus, and glucose, triglycerides, and cholesterol were estimated. For IPGTT and PTT animals were fasted overnight whereas fasting time before ITT was 4 hrs. The figure shows body weight after 12 week HFD feeding (A), plasma glucose (B), triglycerides (C), total cholesterol (D), IPGTT (E), AUC of IPGTT (F), ITT (G), AUC of ITT (H), PTT (I) and AUC of PTT (J). Data are expressed as mean ±SD. (N=6,18), *p<0.05, **p<0.01, ***p<0.001. * vs CON.


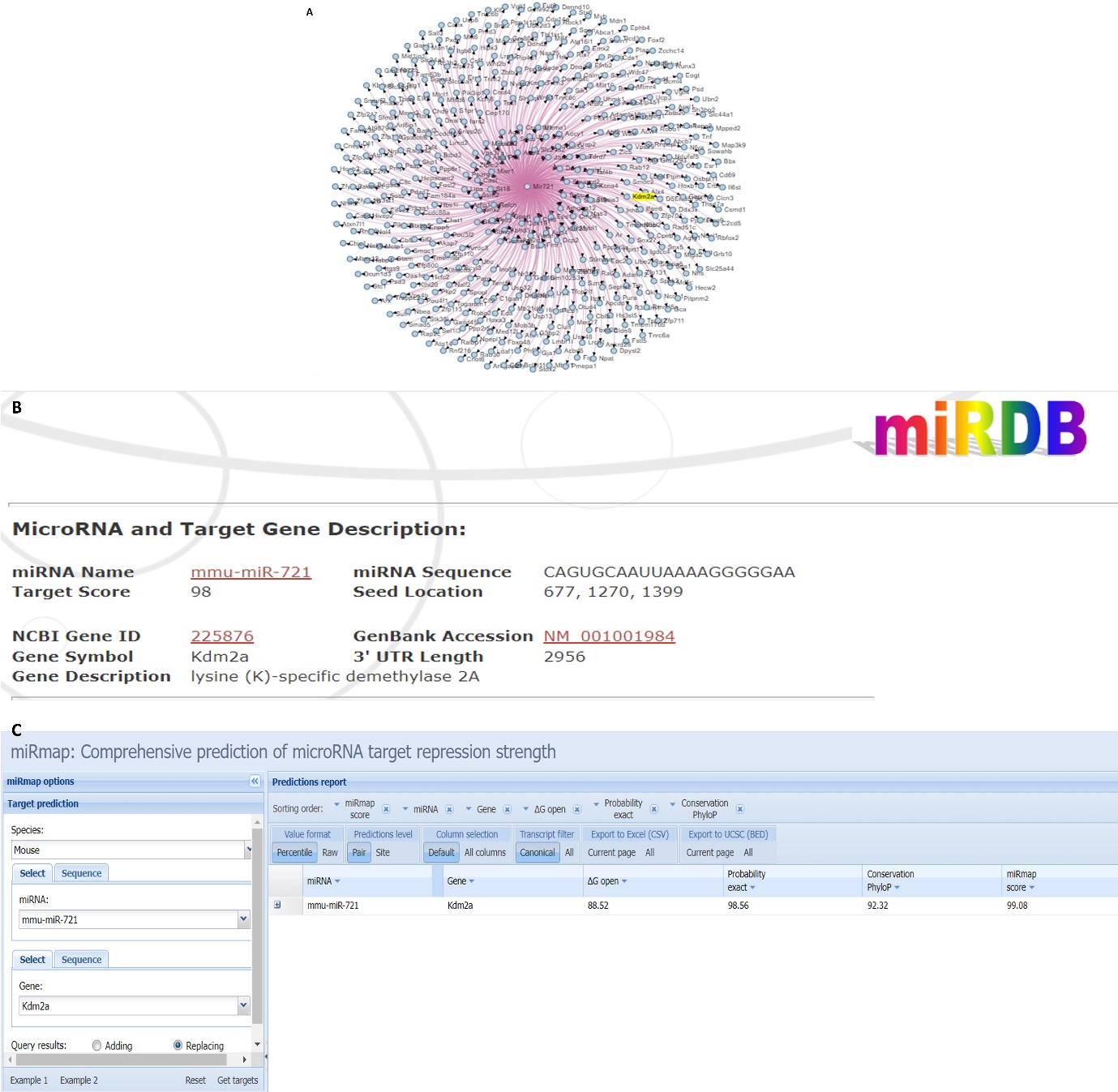


**Fig. S2. Insilico analysis of mm-miR-721 targets.** The figure shows (A) miR-721 target prediction analysis performed at [www.informatics.jax.org/](http://www.informatics.jax.org/) , (B) miR-721 interaction details obtained from miRDB.org and (C) miR-721 targeting of Kdm2A as predicted by miRmap.
